# Cell-Specific Thioautotrophic Productivity of Epsilon-Proteobacterial Epibionts Associated with *Shinkaia crosnieri*

**DOI:** 10.1371/journal.pone.0046282

**Published:** 2012-10-02

**Authors:** Tomo-o Watsuji, Manabu Nishizawa, Yuki Morono, Hisako Hirayama, Shinsuke Kawagucci, Naoto Takahata, Yuji Sano, Ken Takai

**Affiliations:** 1 Subsurface Geobiology Advanced Research Team (SUGAR), Extremobiosphere Research Program (XBR), Institute of Biogeosciences, Japan Agency for Marine-Earth Science and Technology (JAMSTEC), Yokosuka, Kanagawa, Japan; 2 Precambrian Ecosystem Laboratory, JAMSTEC, Yokosuka, Kanagawa, Japan; 3 Geomicrobiology Group, Kochi Institute for Core Sample Research, JAMSTEC, Nankoku, Kochi, Japan; 4 Atomosphere and Ocean Research Institute, the University of Tokyo, Kashiwa, Chiba, Japan; Uppsala University, Sweden

## Abstract

In this study, we report experimental evidence of the thioautotrophic activity of the epibiotic microbial community associated with the setae of *Shinkaia crosnieri*, a galatheid crab that is endemic to deep-sea hydrothermal systems in the Okinawa Trough in Japan. Microbial consumption of reduced sulfur compounds under *in situ* hydrostatic and atmospheric pressure provided evidence of sulfur-oxidizing activity by the epibiotic microbial community; the rate of sulfur oxidation was similar under *in situ* and decompressed conditions. Results of the microbial consumption of reduced sulfur compounds and tracer experiments using ^13^C-labeled bicarbonate in the presence and absence of thiosulfate (used as a thioautotrophic substrate) convincingly demonstrated that the epibiotic microbial community on *S. crosnieri* drove primary production via an energy metabolism that was coupled with the oxidation of reductive sulfur compounds. A combination of tracer experiments, fluorescence *in situ* hybridization (FISH) and nano-scale secondary ion mass spectrometry (Nano-SIMS) indicated that the filamentous cells of the genus *Sulfurovum* belonging to the class *Epsilonproteobacteria* were thioautotrophs in the epibiotic community of *S. crosnieri.* In conclusion, our results strongly suggest that thioautotrophic production by *Sulfurovum* members present as the epibiotic microbial community play a predominant role in a probable nutritional ectosymbiosis with *S. crosnieri.*

## Introduction

Many species of invertebrates that dwell in deep-sea hydrothermal vents are known to host bacteria (epibionts) that adhere to the surface of specialized tissues such as the dorsal setae of the polychaete *Alvinella pompejana*, the gill chamber of the shrimp *Rimicaris exoculata*, the setae of the crabs *Shinkaia crosnieri* and *Kiwa hirsuta*, and the iron-sulfide-coated scales of the scaly-foot snail [1]–[5]. The epibiotic microbial community associated with the host animals commonly includes major phylotypes belonging to the genus *Sulfurovum* within the class *Epsilonproteobacteria* [1]–[5]. Microscopic fluorescence in situ hybridization (FISH) has revealed that members of the genus *Sulfurovum* dominate epibiotic communities and have similar morphological features, such as long and thick filaments [1], [4], [5].

Potential molecular evidences of chemolithotrophic primary production by the epibionts have been obtained by metagenomic characterization of the epibiotic *A. pompejana* community, which is dominated by typical filamentous *Sulfurovum* epibionts [1], [6]. The genes that are involved in the complete reductive tricarboxylic acid (rTCA) cycle as well as in the oxidation of reduced sulfur compounds and hydrogen have been identified in gene assemblages of epsilonproteobacterial epibionts [6]. Similarly, genes that are involved in the rTCA and Calvin cycles as well as in the oxidation of reduced sulfur compounds and hydrogen have been identified in genomic DNA extracted from the epibiotic *R. exoculata* community [7]. These results suggest that the epibiotic microbial community associated with *A. pompejana* and *R. exoculata* mediates functionally active chemoautotrophy via sulfur and/or hydrogen oxidation. In our previous study, we reported clear evidence of autotrophy among the epibiotic microbial community associated with *S. crosnieri*, which predominately comprised morphotypes and phylotypes of members of the genus *Sulfurovum* [5]. We showed that ^13^C-labeled bicarbonate was incorporated in the epibiotic microbial community, and that the primary production by the epibionts was enhanced by the addition of potential thioautotrophic substrates such as sulfide and thiosulfate, but not hydrogen. Although these findings strongly suggest that the epibiotic microbial community associated with *S. crosnieri* harbors functionally active thioautotrophic populations, the thioautotrophic phylotypes have not yet been specified and the expected sulfur-oxidizing activity has not been confirmed [5]. Physiological, genomic, and biochemical characterizations of several *Sulfurovum* isolates have pointed that most of *Sulfurovum* members are chemolithoautotrophs that are sustained by various energy metabolisms that use reduced sulfur compounds [8]–[10]. However, it is not entirely clear whether the long and thick filamentous *Sulfurovum* epibionts associated with these deep-sea vent invertebrates serve as thioautotrophic primary producers.

Therefore, in this study, we attempted to obtain direct evidence of the sulfur-oxidizing activity of the epibionts by investigating the consumption of reduced sulfur compounds by the epibiotic microbial community on *S. crosnieri*. The sulfur-oxidizing activity of the epibiotic microbial community on living *S. crosnieri* individuals and cut setae was characterized under atmospheric and *in situ* hydrostatic pressure to consider the effect of hydrostatic pressure on epibiotic microbial functioning. Moreover, we determined the cell-level-chemoautotrophic productivity of the filamentous *Sulfurovum* epibionts of *S. crosnieri* using a combination of FISH and Nano-SIMS to obtain direct evidence of thioautotrophic activity of the *Sulfurovum* epibionts.

## Results and Discussion

**Figure 1:**
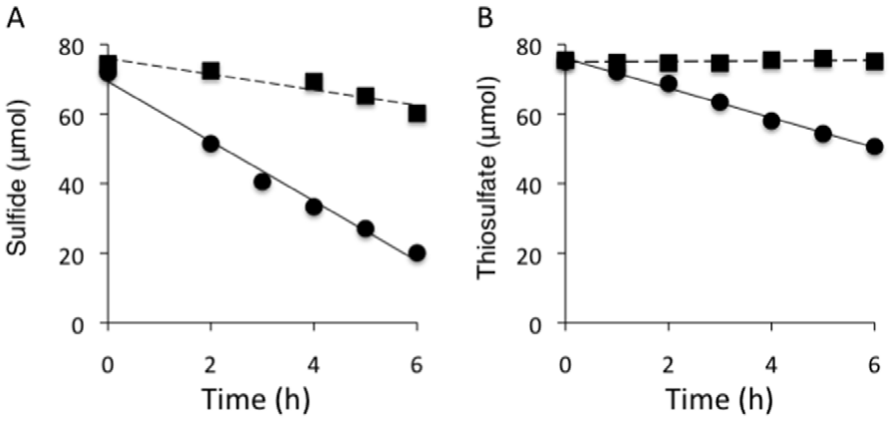
Time course of the consumption of reduced sulfur compounds by epibiotic microbial community associated with *S. crosnieri setae*. The concentration of hydrogen sulfide was examined at the indicated intervals in the absence (black square) and presence (black circle) of the mixed setae samples (A). The unbroken line indicates linear regression analysis for the absence of the mixed setae (*y = *−2.236*x*+75.9, *R^2^* = 0.89) and the solid line indicates linear regression analysis for the presence of the mixed setae (*y = *−8.602*x*+69.4, *R^2^* = 0.99) (A). The concentration of thiosulfate was determined at indicated intervals in the absence (black square) and presence (black circle) of mixed setae samples (B). The unbroken line indicates linear regression analysis for the absence of the mixed setae (*y = *0.079*x*+75.0, *R^2^* = 0.11) and the solid line indicates linear regression analysis for the presence of the mixed setae (*y = *−4.264*x*+76.0, *R^2^* = 0.99) (B). Statistically significant differences in consumption in the presence and absence of the setae samples at individual time points were determined by unpaired t-test (A, P = 2×10^−7^, B, P = 4×10^−10^).

The sulfur-oxidizing activity of mixed setae samples was directly investigated by a time course evaluation of the consumption of sulfide or thiosulfate by the epibionts (Figure 1). The concentration of sulfide decreased under aerobic conditions both in the presence and absence of setae sample, and this was probably due to chemical oxidation by oxygen (Figure 1A). However, a greater concentration of sulfide was consumed during incubation with the mixed setae sample, and the net sulfide consumption rate of the sample was estimated to be 374 µmol· h^−1^·g^−1^ dry weight of setae (Figure 1A). The concentration of thiosulfate decreased only on incubation with the setae sample (Figure 1B). The estimated net rate of consumption of thiosulfate in the mixed setae sample was 99 µmol·h^−1^·g^−1^ dry weight of setae (Figure 1B). Our results clearly indicated that epibiotic microbial community of the *S. crosnieri* setae contained sulfur-oxidizing populations although it is unclear whether the populations were autotrophic and/or heterotrophic. This is the first direct evidence for the existence of functionally active sulfur-oxidizing populations in the epibiotic microbial community associated with invertebrates that inhabit deep-sea hydrothermal vents.

The sulfur-oxidizing activity of the epibionts associated with living *S. crosnieri* individuals was investigated under atmospheric and elevated hydrostatic pressure using a newly developed high-pressure continuous-flow apparatus (Figure S1). The hydrostatic pressure was increased to 12.0 MPa, since this value corresponds to the hydrostatic pressure at a water depth of approximately 1200 m. The depth of the major *S. crosnieri* habitats, such as the Iheya North and Hatoma Knoll fields, ranges from approximately 1000 to 1500 m [5], [11]. The *S. crosnieri* individuals were confirmed to be alive during incubation in both piezophilic and non-piezophilic conditions, since we were able to observe them moving and performing a number of behaviors. A decrease in the sulfide concentration in the effluent seawater was detected under all conditions, and this was probably due to chemical oxidation by oxygen (Table 1). However, greater sulfide consumption rates were obtained in the incubation experiments performed with living *S. crosnieri* individuals (Table 1). The net sulfide consumption rate of the living individuals was estimated to be 576 and 557 µmol·h^−1^·g^−1^ dry weight of setae under 0.1 and 12.0 MPa, respectively (Table 1). Although we had expected that the sulfide consumption rate of the *S. crosnieri* epibionts would potentially be affected by hydrostatic pressure, these results show that the sulfur-oxidizing activity of the epibionts was similar under conditions mimicking *in situ* hydrostatic pressure and ambient surface pressure (Table 1). Several piezophilic thioautotrophs have been isolated from deep-sea hydrothermal environments, and their growth rates have been shown to be strongly affected by hydrostatic pressure [12]. Since growth rate is regulated by whole cellular functions, it may be much more responsive to differences in hydrostatic pressure than the energy and carbon metabolism pathways that involve sulfide oxidation. This is the first study to demonstrate that the sulfur-oxidizing activity of an epibiotic microbial community is not significantly affected by change in hydrostatic pressure. These results can be interpreted as indicating that the metabolic rates of epibiotic microbial communities associated with invertebrates from deep-sea vents are not strongly influenced by depressurization, and that the rate constants determined under non-pressurized conditions can be applied to estimations of function and metabolism *in situ* in deep-sea habitats.

**Table 1:** Net consumption rates of sulfide during continuous supply of sulfide to a living *S. crosnieri* individual at a hydrostatic pressure of 0.1 and 12.0 MPa.

Pressure	Sulfide consumption rate (µmol/h)		Net sulfide consumption rate	
(MPa)	with individual	without individual	(µmol/h/individual)	(µmol/h/g of setae)
0.1	51.7	12.7	39.0	576
12.0	52.1	12.9	39.2	557

The comparison of the sulfide consumption rates of the epibionts associated with living individual and dissected setae showed similar levels of slightly different sulfur-oxidizing activities (Figure 1 and Table 1). The net sulfide consumption rate of the epibionts associated with living *S. crosnieri* individual (576 µmol·h^−1^·g^−1^ setae) was 1.5-fold higher than that associated with dissected setae (383 µmol·h^−1^·g^−1^ setae; Figure 1A and Table 1). The difference may simply be explained by the subsidiary functions of microbial communities populating the gut and body surface or as an analytical deviation resulting from our limited number of measurement points. However, the rate of sulfide consumption may actually differ between the epibionts associated with living individual and those associated with dissected setae. It is likely that the convective transport of sulfide to the epibionts associated with living *S. crosnieri* individuals differs from those associated with dissected setae. In our experiments investigating sulfur-oxidizing activity, living *S. crosnieri* individuals were frequently observed to move in glass vessels during incubation whereas dissected setae remained in a static position in artificial seawater in a glass bottle. It is difficult to assume that there were significant differences in the physiological state and abundance of the epibiotic microbial community associated with living individual and dissected setae because the sulfide consumption experiments were conducted using fresh *S. crosnieri* individuals and setae are always covered with the numerous epibionts. Therefore, the different consumption rates suggest that the epibiotic microbial community associated with living *S. crosnieri* is advantageous for ensuring accessibility to sulfide. This may be an important insight into how epibiotic microbial communities on deep-sea vent invertebrates benefit from their hosts.

The autotrophic productivity of the epibiotic microbial community associated with the mixed setae was determined by incubation with NaH^13^CO_3_ in the presence and absence of thiosulfate (used as a sulfur-oxidizing compound) (Table 2). After 48 h of incubation, the bulk organic carbon of the epibiotic microbial community was found to be enriched with ^13^C during incubation in the presence and absence of thiosulfate (Table 2). The ^13^C enrichment of bulk organic carbon in the epibiotic microbial community was 2.4-fold higher (unpaired t-test, P = 4×10^−5^) during incubation with thiosulfate compared with during incubation without thiosulfate (Table 2). Although previous uptake experiments using ^13^C-labeled tracers and a previous analysis of potentially functional genes suggested that sulfur-oxidizing chemoautotrophic production occurs in such epibiotic microbial communities [5]–[7], the result of a combination of thiosulfate-enhanced enrichment of ^13^C-labeled bicarbonate and sulfur-oxidizing activity in similar subsamples of mixed setae convincingly demonstrated presence of a thioautotrophic population in the *S. crosnieri* epibionts (Figure 1 and Table 2).

**Table 2:** Stable carbon isotope analysis of the incorporation of ^13^C-labeled bicarbonate by the epibiotic microbial community on *S. crosnieri* setae.

Incubation Time (hours)	Tracer and Additional Energy Source	δ^13^C (‰)*	Average ^13^C/^12^C	% Enrichment
0	–	−41.3±0.2	0.01077±0.00002	–
48	^13^CO_2_	778±34	0.0200±0.0004	74±3
	^13^CO_2_ + thiosulfate	1851±68	0.0320±0.0008	176±7

* **δ** ^13^C values were measured in triplicate and were expressed as the mean ± SD.

** The ^13^C enrichment E (%) was calculated using the following equation: E = (F_sample_/F_control_ − 1) ×100 (%). F_sample_ and F_control_ represent the isotopic abundance of ^13^C atoms of the epibionts before and after ^13^C labeling [F = (^13^C/^12^C)/(1+^13^C/^12^C)].

**Figure 2:**
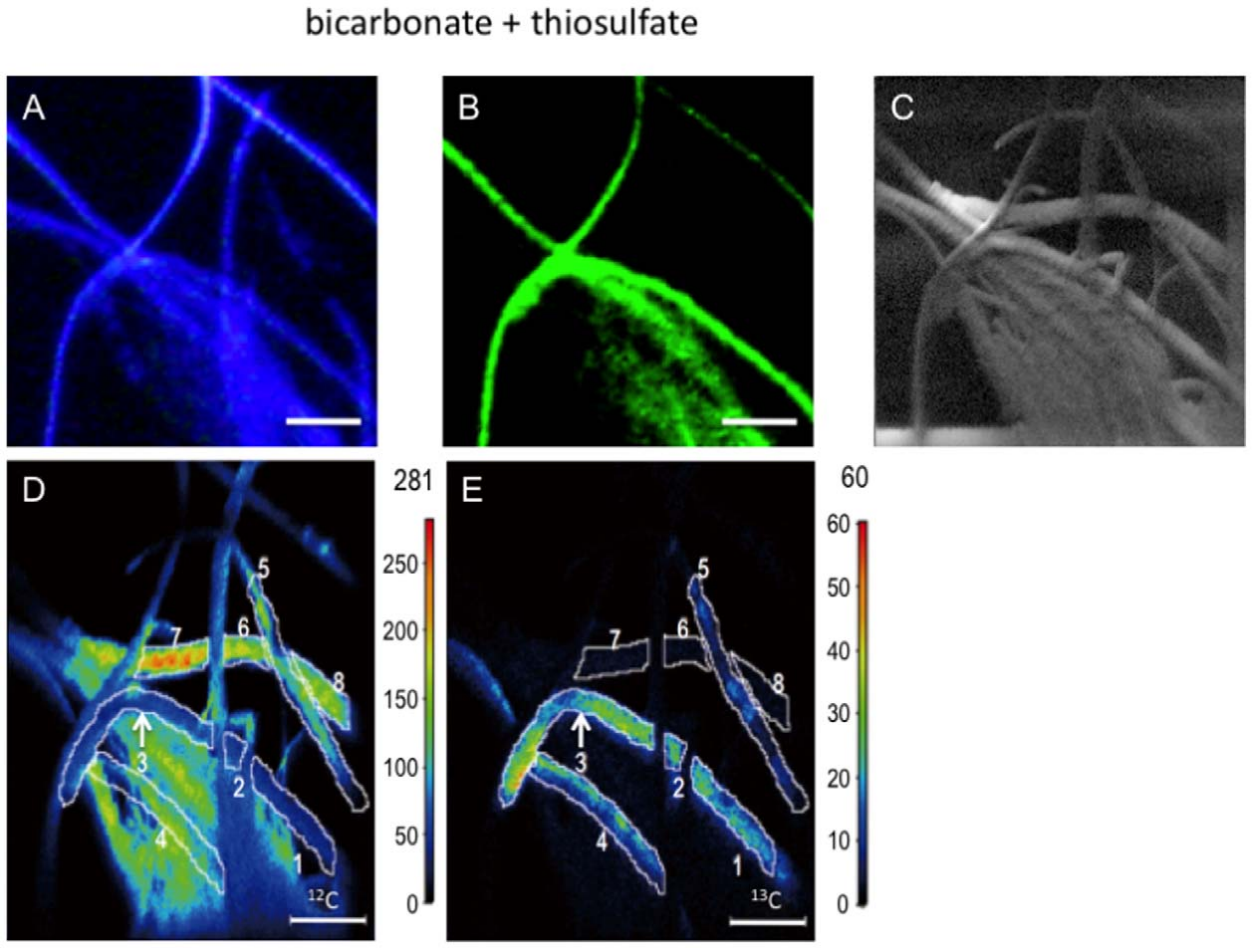
Analyses of epibiotic microbial cells after ^13^C bicarbonate tracer experiments. Microscopic image of DAPI-stained epibiont cells in a specimen (A). Microscopic image of epibiont cells specifically bound to the EPI653 probe, which indicates that members of the genus *Sulfurovum* belonging to the class *Epsilonproteobacteria* were present in the same specimen (B). Scanning electron micrograph of epibiont cells in the same specimen (C). ^12^C-mapping image of epibiont cells in the same specimen performed using Nano-SIMS (D). ^13^C-mapping image of epibiont cells in the same specimen performed using Nano-SIMS (E). The values (secondary ion counts) corresponding to each of the colors are shown in the scale to the right of each map (D and E). The estimated ^13^C/^12^C ratios of the cells that are enclosed by the white lines are indicated in Table 3. The scale bar is 10 µm.

**Figure 3:**
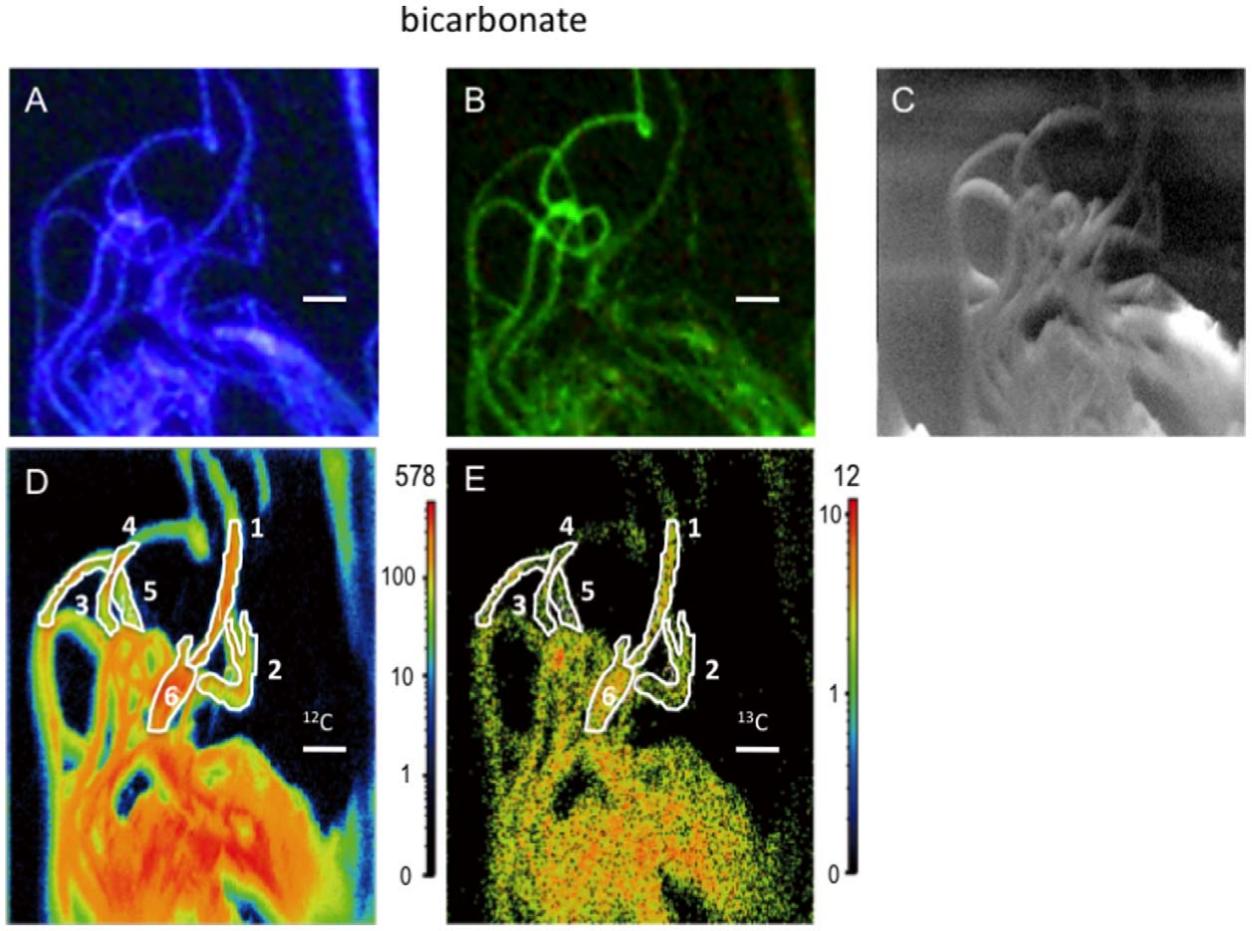
Analyses of epibiont cells after ^13^C bicarbonate tracer experiments performed in the presence of potential thioautotrophic substrates. Microscopic image of DAPI-stained epibiont cells in a specimen (A). Microscopic image of epibiont cells specifically bound to the EPI653 probe, which indicates that members of the genus *Sulfurovum* belonging to the class *Epsilonproteobacteria* were present in the same specimen (B). Scanning electron micrograph of epibiont cells in the same specimen (C). ^12^C-mapping image of epibiont cells in the same specimen performed using Nano-SIMS (D). ^13^C-mapping image of epibiont cells in the same specimen performed using Nano-SIMS (E). The values (secondary ion counts) corresponding to each of the colors are shown in the scale to the right of each map (D and E). The estimated ^13^C/^12^C ratios of the cells that are enclosed by the white lines are indicated in Table 3. The scale bar is 20 µm.

After ^13^C-bicarbonate tracer experiments in the presence and absence of thiosulfate, subsamples of mixed setae were used for FISH and Nano-SIMS to clarify the cell-specific trophic nature of the *Sulfurovum* epibionts (Figures 2 and 3). FISH using a *Sulfurovum*-specific probe demonstrated that the predominant morphotype of the epibionts exhibited long and thick filaments, and these were identified as the *Sulfurovum* members within the *Epsilonproteobacteria* (Figures 2B and 3B). FISH–Nano-SIMS of the epibiotic microbial community after ^13^C-bicarbonate incubation with additional thiosulfate indicated that a *Sulfurovum* species that contributed to ^13^C enrichment of cellular carbon was present (Figure 2B, 2D and 2E). The carbon isotope abundance in representative locations of the epibiont cells was quantified, and the estimated average ^13^C/^12^C ratios were compared to the value of the bulk organic carbon of the epibiotic microbial community before the tracer experiments (control epibiont cells) (Figure 2 and Tables 2 and 3). The ^13^C/^12^C ratios of the filamentous *Sulfurovum* cells were enriched up to 2713% compared to the average ^13^C/^12^C ratio of the control epibiont cells (Figure 2 and Table 3). Nano-SIMS carbon isotope mappings (^12^C and ^13^C) of the epibionts incubated in the absence of thiosulfate showed very similar distribution patterns of ^12^C and ^13^C, indicating that all epibionts were either enriched or not enriched with ^13^C (Figure 3). Comparison with epibiont cells before tracer experiments indicated that the representative *Sulfurovum* epibionts displayed no cellular carbon ^13^C enrichment during the tracer experiments performed in the absence of thiosulfate (Table 3). These results indicate that filamentous *Sulfurovum* cells assimilated inorganic carbon only after incubation in the presence of thiosulfate (unpaired t-test, P = 3×10^−6^), whereas our previous study revealed that the epibiotic microbial communities on *S. crosnieri* assimilated inorganic carbon both in the presence and absence of reduced sulfur compounds [5]. In conclusion, it became evident that thiosulfate served as an energy source for autotrophic production by the epibionts. Therefore, FISH–Nano-SIMS of the epibiotic microbial community during ^13^C-bicarbonate incubation provided clear evidence of the thioautotrophic productivity of the *Sulfurovum*-affiliated epibionts, and strongly suggested that the predominant epibiotic population comprised sulfur-oxidizing chemoautotrophs that were capable of using reduced sulfur compounds as an energy sources. This study is the first report of cell-specific thioautotrophic metabolism in epibiotic microbial communities associated with deep-sea vent invertebrates. Autotrophy of the microbial communities associated with deep-sea and terrestrial invertebrates has been demonstrated previously by RuBisCO activity, gene expression of ATP citrate lyase (a key enzyme in the rTCA cycle), and incorporation of labeled bicarbonate [13]–[15]. In addition, our previous study indicated the possible nutritional transportation from the organic carbons produced by chemoautotrophic and methanotrophic epibionts on *S. crosnieri* to the host body because ^13^C-labeled bicarbonate and methane were incorporated into the *S. crosnieri* body as well as the epibiotic microbial community [5]. From our previous and present studies, it is concluded that primary production by sulfur-oxidizing chemoautotrophs as well as methanotrophs in the epibiotic microbial community associated with *S. crosnieri* provide nutritional support for the host, and that *Sulfurovum* members, which constitute a large proportion of the epibiotic community, play a predominant role in the probable nutritional ectosymbiosis with *S. crosnieri*.

**Table 3:** Cell-specific incorporation of ^13^C-labeled bicarbonate by the *Sulfurovum* epibionts on *S. crosnieri* seta in the presence and the absence of thiosulfate according to FISH–Nano-SIMS.

Tracer	Location	Average ^13^C/^12^C	Enrichment (%)
	1*	0.428±0.086	2713±588
	2*	0.292±0.058	2019±435
NaH^13^CO_3_	3*	0.394±0.079	2551±552
+	4†	0.135±0.027	1012±225
thiosulfate	5†	0.082±0.016	608±143
	6†	0.010±0.002	−10±20
	7†	0.011±0.002	−5±20
	8†	0.010±0.002	−10±20
	1*	0.009±0.002	−15±20
	2*	0.010±0.002	−10±20
NaH^13^CO_3_	3*	0.011±0.002	−5±20
	4*	0.009±0.002	−15±20
	5*	0.009±0.002	−15±20
	6*	0.010±0.002	−10±20
–	Control epibiont cells	0.01077	–

The percent enrichment was estimated using the average ^13^C/^12^C ratio of bulk organic carbon of the epibiotic microbial community before the experiment shown in Table 2.

* The filamentous cells were identified as *Sulfurovum* members by FISH.

† The filamentous cells were not identified as the *Sulfurovum* members by FISH.

FISH–Nano-SIMS indicated that cellular ^13^C enrichment did not occur among the *Sulfurovum* epibionts during the tracer experiments performed in the absence of thiosulfate (Figure 2 and Table 3), whereas ^13^C enrichment of bulk organic carbon in the epibiotic microbial community was detected after incubation in the absence of thiosulfate (Table 2). In our previous study [5], it was hypothesized that ^13^C-labeled bicarbonate enrichment of the epibiotic community without any additional energy source was attributed to the potential metabolic activities of epibionts using the remaining reduced sulfur compounds that might be preserved inside and/or outside the epibiont cells. Indeed, in the microbial epibiotic community associated with *R*. *exoculata*, sulfur granules are only observed inside thin filamentous epibiont cells, while they are absent from thick filamentous epibionts [16]. Therefore, it was inferred that ^13^C-labeled bicarbonate incorporation that was independent of any additional energy source might be promoted by chemoautotrophic populations of epibionts (e.g., potentially thioautotrophic gamma-proteobacterial populations with thin filamentous morphotypes) other than thick filamentous *Sulfurovum* epibionts [5]. Since the short and thin filamentous morphotypes of gamma-proteobacterial epibionts are usually located deep within aggregates of the long filamentous morphotypes of *Sulfurovum* epibionts [5] and the primary beam did not reach the interior populations of epibionts in Nano-SIMS analysis, FISH–Nano-SIMS did not examine the potential metabolic activities of gamma-proteobacterial epibionts using the remaining reduced sulfur compounds.

In this study, the thioautotrophic productivity of the predominant *Sulfurovum* epibionts was verified using cell-specific FISH–Nano-SIMS. However, the thioautotrophic activity of the epibiotic microbial community requires further investigation. In cell-specific FISH–Nano-SIMS, the filamentous epibionts that did not bind to the *Sulfurovum*-specific FISH probe showed considerable ^13^C enrichment of cellular carbon during incubation in the presence of thiosulfate compared with *Sulfurovum* epibionts incubated in the absence of thiosulfate (unpaired t-test, P = 0.0002; Figure 2A, 2B, 2E and Table 3). These results show that the epibiotic microbial community of *S. crosnieri* setae includes the *Sulfurovum* members and currently unidentified thioautotrophs. In addition, not all of the *Sulfurovum* filamentous epibionts showed significant enrichment of ^13^C-carbon during incubation in the presence of thiosulfate (Figure 2B, 2E). This may indicate that not all the *Sulfurovum* filamentous epibionts possess the dissimilatory thiosulfate oxidation pathway coupled with carbon fixation or express the enzymes that are involved in dissimilatory thiosulfate oxidation. This is also one possible explanation for why the consumption rate of sulfide was greater (4-fold) than that of thiosulfate (Figure 1). Whole genome analysis [9] and biochemical characterization [17] have indicated that the quinone reductase (Sqr) and epsilon-type Sox system are potentially responsible for sulfide, polysulfide, and thiosulfate oxidation to sulfate in the chemolithotrophic energy metabolism of *Sulfurovum* sp. strain NBC37-1. Although *Sulfurovum* sp. strain NBC37-1 constitutively expresses these sulfur-oxidizing enzymes, it remains unclear whether other *Sulfurovum* epibionts always express the enzymes that are involved in thioautotrophic metabolism. A previous study revealed that the *Sulfurovum* members and their relatives are not necessarily thioautotrophic chemoautotrophs, and that some represent strictly hydrogenotrophic chemoautotrophs [8]. The long and thick filamentous *Sulfurovum* epibionts on *S. crosnieri* setae morphologically appear homogenous, but considerable genetic heterogeneity is perceived in the 16S rRNA gene phylotype composition [5]. Although the *Sulfurovum* epibionts seem to be primarily thioautotrophs, the long and thick filamentous *Sulfurovum* epibionts may consist of metabolically heterogeneous components. These points will be the focus of further investigations.

## Materials and Methods

### Ethics Statement

The locations for sample collection were not privately owned or protected in any way and no specific permits were required for the described field studies and sample collection. The field studies did not involve any endangered or protected species.

### Collection of *S. crosnieri* from the Deep-sea Hydrothermal Field

*S. crosnieri* individuals were collected from the Iheya North field in the Okinawa Trough, Japan, during dive#1062 on October 9, 2009 (27° 47.46′N, 126° 53.01′E, depth 1004 m) and dive#1355 on March 19, 2012 (27° 47.46′N, 126° 53.81′E, depth 986 m) using the JAMSTEC remotely operated vehicle (ROV) “*HyperDolphin*”. Individuals were collected using a suction sampler and were stored in a box of chilled water in the ROV. Immediately after onboard recovery, the individuals were thoroughly washed using sterile artificial seawater. After washing, setae from the three individuals collected during dive#1062 were cut and mixed together. These procedures were performed below 5°C because the ambient temperature of *S. crosnieri* in their natural habitat ranges from 4 to 6°C. Mixed setae collected during dive#1355 were used for measuring sulfur-oxidizing activity and tracer experiments with ^13^C-labeled bicarbonate. Two living *S. crosnieri* individuals (37-mm carapace length) collected during dive#1355 were used for measuring sulfur-oxidizing activity under both *in situ* hydrostatic (12.0 MPa) and atmospheric pressure (0.1 MPa).

### Sulfur-oxidizing Activity Measurements

Mixed setae samples from the three individuals were used for measuring sulfur-oxidizing activity; measurement was performed onboard. The mixed setae were incubated at 5°C in 295-mL glass bottles that were sealed with butyl rubber stoppers and contained 200 mL of artificial seawater (25 g·L^−1^ NaCl, 4.2 g·L^−1^ MgCl_2_·6H_2_O, 3.4 g·L^−1^ MgSO_4_·7H_2_O, 0.5 g·L^−1^ KCl, 0.7 g·L^−1^ CaCl_2_·2H_2_O, 14 mg·L^−1^ K_2_HPO_4_, 2.1 mg·L^−1^ NH_4_Cl and 6.8 mg·L^−1^ NaNO_3_; adjusted to pH 7.3) filtered using a 0.22 µm-pore membrane under air. The artificial seawater was supplemented with sodium sulfide and sodium thiosulfate (used as reduced sulfur compounds) in the presence of 1 mM sodium bicarbonate at final concentrations of 375 µM. The artificial seawater in the bottles was subsampled at the indicated intervals. The dry weight of the mixed setae incubated with sodium sulfide and sodium thiosulfate was 17 and 43 mg, respectively.

The sulfur-oxidizing activity of *S. crosnieri* setae was examined using a high-pressure continuous-flow apparatus at 5°C; this supplied a certain concentration of sodium sulfide and oxygen to a living *S. crosnieri* individual confined in a glass incubation vessel under a given hydrostatic pressure (up to 25 MPa) (Figure S1). The apparatus comprised a plunger pump (LC-10Ai, Shimadzu Co., Japan), a stainless steel pressure chamber with two observation windows (Horiguchi Ironworks Co., Japan), and glass incubation vessels (Horiguchi Ironworks Co., Japan) directly connected with hydraulic tubes to the liquid supplied by the plunger pump and discharged through a pressure relief valve (Figure S1). All the hydraulic tubing for transportation of the liquid was manufactured using PEEK or silica-coated stainless steel (Swegelok). The hydrostatic pressure (up to 25 MPa) and the continuous flow (up to 9.99 mL·min^−1^) in the glass incubation vessels were regulated by the plunger pump and the pressure relief valve when the experiments were conducted under high hydrostatic pressure (Figure S1). If the experiments were conducted under atmospheric pressure (0.1 MPa), the pressure relief valve was disconnected. The hydrostatic pressure (up to 25 MPa) of the tap water used to fill the stainless steel pressure chamber was adjusted by a hydraulic pump (HP-150, Syn Co., Kyoto, Japan). The cylinder glass incubation vessel used as a specimen holder (diameter, 50 mm; length, 80 mm; volume, 182 mL) had a stainless steel cover with inlet and outlet ports that were sealed by a butyl rubber gasket (thickness, 5 mm) at the top and butyl rubber bellows (thickness, 5 mm) at the bottom (Figure S2). The butyl rubber bellows served as a pressure buffer for the liquid between the inside of the glass vessel and the pressure chamber. During the incubation experiments in which living animals were used, the motility and behaviors of the animals were monitored through the observation windows in the pressure chamber as the specimen holder was made of cylinder glass (Figure S2).

In experiments measuring the sulfur-oxidizing activity of epibionts associated with living *S. crosnieri* individuals, artificial seawater supplemented with 270 µM sodium sulfide and 1 mM sodium bicarbonate was supplied at a flow rate of 9.0 mL·min^−1^ for 60 min at 5°C under hydrostatic pressure of 0.1 or 12.0 MPa. Pressurization to 12.0 MPa required 1–2 min. The influent and effluent seawater were sampled at 10-min intervals, and the sulfide concentration was determined as described below. In both the piezophilic and non-piezophilic experiments, the sulfide concentration became constant after 40 min of incubation. We used the sulfide concentrations of the influent and effluent seawater at 50 and 60 min after the initiation of continuous flow to determine the average consumption rate of sulfide. The dry weight of *S. crosnieri* setae used for sulfur-oxidizing activity experiments under 0.1 and 12.0 MPa was 68 and 70 mg, respectively.

The concentration of sulfide in the seawater was determined using the methylene blue method [18]. The concentration of thiosulfate was determined using ion chromatography using the Shim-pack IC-A3 column (Shimadzu Co., Japan) and a buffer system consisting of 8 mM *p*-hydroxybenzoic acid, 3.2 mM bis(2-hydroxyethyl)imino-tris(hydroxymethyl)methane, and 50 mM boric acid.

### Tracer Experiments

The mixed setae samples were also used for the tracer experiments. The mixed setae were incubated for 48 h at 5°C in 295-mL glass bottles sealed with butyl rubber stoppers and containing 200 mL of artificial seawater under air. Supplemental ^13^C-labeled bicarbonate (NaH^13^CO_3_) was added to the seawater at a final concentration of 1 mM in the presence or absence of 300 µM sodium thiosulfate, which was used as a reduced sulfur compound. NaH^13^CO_3_ was purchased from Cambridge Isotope Laboratories, Inc. (Andover, MA, USA). After 48 h of incubation onboard, the mixed setae samples were harvested and frozen at −80°C. At the onshore laboratory, the setae samples were lyophilized and the epibiont cells were used for ^13^C composition analysis.

### FISH

After the tracer experiments, a portion of the mixed setae samples was fixed using 4% paraformaldehyde in phosphate-buffered saline overnight at 4°C and stored in 50% ethanol in phosphate-buffered saline at −30°C until FISH. The fixed samples were then spotted onto glass slides and air dried before dehydration by sequential washes in 50%, 80%, and 100% (v/v) ethanol for 3 min each. Hybridization with the probe EPI653, which was specifically designed for detecting *Sulfurovum* phylotypes within the class *Epsilonproteobacteria* [5], was performed as previously described [5]. The probe was labeled with Alexa 488. After hybridization, the epibiotic microbial community associated with the setae was transferred to a brass pedestal (Vector Laboratories, Burlingame, CA, USA) and viewed using a Nikon E800 epifluorescence microscope (Nikon Instruments, Inc., Melville, NY, USA). The images were collected and analyzed using NIS Elements AR 2.30 and Hotfix (Build 312) image analysis software (Nikon Instruments, Inc.).

### Nano-scale Secondary Ion Mass Spectrometry

Cell-specific carbon isotope ratios of the epibiotic microbial community associated with *S. crosnieri* setae were measured using the NanoSIMS 50 ion microprobe (AMETEK Co., CAMECA, Courbevoie, France) at the University of Tokyo. The lateral resolution of Nano-SIMS is up to 50 nm, and it has been successful in determining the isotopic distributions of carbon in cell and mineral samples at the submicron scale and at diameters of a few microns [19], [20]. Following incubation with NaH^13^CO_3_ in the presence or absence of thiosulfate, the subsamples were prepared for FISH and placed on 10-nm thick brass plates that were gold coated for charge compensation. Before carbon isotopic measurement, Cs^+^ ions were implanted on the sample surface under a high current beam (15 pA·s·µm^−2^). Carbon isotopic measurement was performed using a 6-pA Cs^+^ primary beam that was stepped over the 100×100 µm field of a 256×256 pixel raster with a counting time of 1 or 5 ms per pixel.

Negative secondary ions (^12^C^−^, ^13^C^−^, ^12^C^12^C^−^, and ^12^C^13^C^−^ ions) were accelerated by the application of 8 kV at the sample surface and were simultaneously detected using four electron multipliers in the ion-counting mode at a mass resolution that was sufficient to separate ^13^C^−^ from ^12^CH^−^ and ^12^C^13^C^−^ from ^12^C^12^CH^−^. The entrance slit width was set to approximately 30 µm, and the exit slit width of each EM detector was set to 50 µm.

Recorded images and data were processed using CAMECA WinImage software. The different scans of each image were aligned to correct for image drift during acquisition. ^13^C enrichment of a microbial cell was calculated by drawing a region of interest (ROI) on C^−^ images and calculating the ^13^C/^12^C ratio from ^13^C^−^/^12^C^−^ ratio of the ROI by correcting for the effect of instrumental mass fractionation. The instrumental mass fractionation of C isotopes was estimated to be −35‰ by measuring the ^13^C^−^/^12^C^−^ ratios of epibionts for which the ^13^C/^12^C ratios were determined by the conventional EA-IRMS method. The reproducibility of the ^13^C^−^/^12^C^−^ ratio of an in-house standard (*Escherichia coli*) was 20% (1SD, n = 10). ^13^C enrichment E (%) was calculated with the following equation: E = (F_sample_/F_control_–1)×100 (%) where F_sample_ and F_control_ show the isotopic abundance of ^13^C atoms of the epibionts before and after ^13^C labeling [F = (^13^C/^12^C)/(1+^13^C/^12^C)]. The data were expressed relative to the PDB (Peedee belemnite) standard.

### Stable Carbon Isotopic Analyses of ^13^C-labeled and Non-labeled Setae

The mixed setae samples obtained from the three *S. crosnieri* individuals before and after the ^13^C-labeled tracer experiments were thoroughly washed in sterile artificial seawater without carbonates, frozen, and stored at −80°C. Contaminating carbonates in the samples were removed as previously described [5]. The ^13^C composition of the samples was determined using a mass spectrometer (Delta Plus XP; Thermo Finnigan, Bremen, Germany) that was coupled online via a Finnigan ConFlo III interface with an elemental analyzer (FlashEA 1112; ThermoQuest, Milan, Italy). All samples were measured in triplicate.

## Supporting Information

Figure S1 **High-pressure continuous-flow system.**
(TIF)

Figure S2 **Structure of the specimen holder.**
(TIF)
