# Supplementary figures and images for: Cell-Specific Thioautotrophic Productivity of Epsilon-Proteobacterial Epibionts Associated with Shinkaia crosnieri
Source: PLoS One. 2012 Oct 2;7(10):e46282. doi: 10.1371/journal.pone.0046282 (PMC3462759; doi:10.1371/journal.pone.0046282)

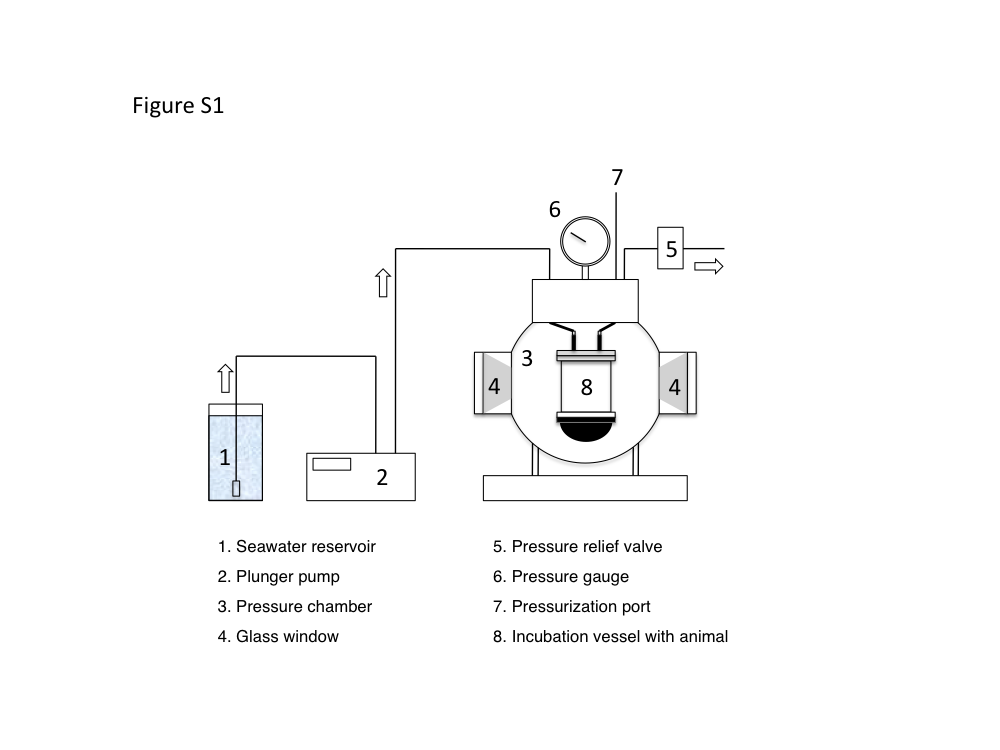

Supplement: Figure S1 — High-pressure continuous-flow system. (TIF) [file pone.0046282.s001.tif]
